# Relationship between fear of COVID‐19 and mental health of Chinese nurses: The mediating effects of psychological capital and burnout

**DOI:** 10.1002/nop2.2136

**Published:** 2024-03-15

**Authors:** Chengxiang Liu, Sainan Li, Juan Zhou, Miao Zhang, Hong Chen

**Affiliations:** ^1^ Department of Anesthesia and Perioperative Medicine The Second Affiliated Hospital of Anhui Medical University Hefei China; ^2^ Department of Nursing The Second Affiliated Hospital of Anhui Medical University Hefei China

**Keywords:** burnout, fear of COVID‐19, mediation, mental health, nurse, psychological capital

## Abstract

**Aim:**

The aim of the study was to investigate the correlation between fear of COVID‐19 and mental health of nurses and the effects of psychological capital and burnout in this relation.

**Design:**

A cross‐sectional study.

**Methods:**

The online surveys were conducted among mainland Chinese nurses. Participants (*n* = 445; average age 32.89 ± 6.76 years) completed an online‐questionnaire based on the Fear of COVID‐19 Scale, the Psychological Capital Scale, Maslach Burnout Inventory Human Services Survey for Medical Professionals Scale and the 12‐Item Short Form Health Survey. Data analysis was conducted by Pearson's correlation analysis, Harman single‐factor test and the bootstrap method for mediating effect testing.

**Results:**

(1) The study demonstrated a significant direct effect of fear of COVID‐19 on nurses' mental health, as well as on mediating factors such as burnout and psychological capital. (2) Regression analyses confirmed that while psychological capital bolstered mental health, burnout undermined it, with fear of COVID‐19 further imposing a negative influence. (3) Fear of COVID‐19 exerted an effect on the mental health of nurses by the independent and chain intermediary functions of psychological capital and burnout, resulting in a total mediating effect of −0.233.

## 1 INTRODUCTION

The SARS‐CoV‐2 virus pandemic (hereafter referred to as the COVID‐19 pandemic) disrupted the daily lives of millions of individuals worldwide. Healthcare workers, particularly nurses, at the forefront of the response to the virus have been significantly affected. Nurses faced numerous challenges, such as providing care to COVID‐19 patients, working long hours, and coping with the psychological effects of the pandemic (Cai et al., 2020).

Investigators uncovered that nurses had a higher likelihood of mental health issues, including but not limited to anxiety disorders, depressive episodes, and post‐traumatic stress disorder (Sampaio et al., 2021). In the prior 3 years, Chinese nurses exhibited a gradual deterioration in their mental well‐being (Varghese et al., 2021). Given that trend, it is imperative that scientific measures be taken to provide mental health guidance and support for this vulnerable population. Lai et al. (2020) reported that the prevalence of severe depression was 7.1% among 764 nurses working in 34 hospitals with specialized fever clinics or COVID‐19 wards. In a separate investigation of 3228 nurses in Sichuan Province and Wuhan City, Zheng et al. (2021) reported a markedly higher depression prevalence of 47.1%. Zhang et al. (2022) reported that the prevalence of anxiety, depression, and post‐traumatic stress disorder was 40%, 46%, and 61%, respectively, among 100 nurses who assisted in Wuhan and who resided in Hangzhou, China. Li et al. (2023) surveyed 138,279 nurses working in 243 hospitals and revealed that a considerable proportion of respondents experienced burnout (34%), anxiety (41.8%), and depression (55.5%). Poor mental health of nurses may have detrimental consequences, such as medical mistakes (Arimura et al., 2010), reduced job satisfaction, compromised decision‐making ability, increased absenteeism, and staff turnover (Pang et al., 2020). The close correlation between mental health and nursing care safety and quality has been extensively investigated (Cheng et al., 2020). Many studies have been performed to scrutinize the diverse factors that influence the psychological well‐being of nurses (De Brier et al., 2020).

**FIGURE 1:**
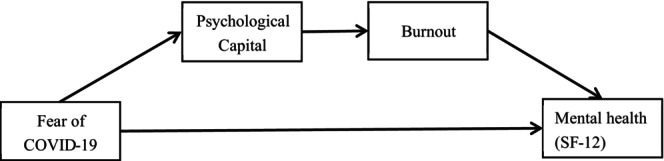
Model of burnout and psychological capital as proposed mediators of the effect of fear of COVID‐19 on nursing staff mental health.

Research on the effect of fear of COVID‐19 on mental health is relatively mature. Şimşir et al. (2022) conducted a meta‐analysis of 62 studies and provided evidence of a significant association between fear of COVID‐19 and mental health problems. Fear can activate the stress response, causing the release of adrenaline and cortisol that can negatively affect health, including deterioration of mental health (Chu et al., 2023; Drigas & Mitsea, 2021). Most research conducted on the determinants of mental health has centred on organizational factors such as workload (Farid et al., 2020) and job demands and the effectiveness of interventions (Maben & Bridges, 2020). There is a paucity of literature about the mechanisms and mediating factors that link fear of COVID‐19 to mental health status. To bridge this research gap, it is imperative to measure the effect of fear of COVID‐19 on the mental health of nurses while also examining the mediating effect of psychological capital between fear of COVID‐19 and burnout, as illustrated in Figure 1. Such a study would not only enrich the theoretical scope of the determinants of mental health among nurses, but also the study would provide evidence to help develop targeted strategies for nurses. These strategies would ultimately be used to address the issue of mental health more effectively among nurses and stabilize the healthcare workforce.

### 1.1 Fear of COVID‐19 effect on nurse mental health

On the basis of the Transactional Model of Stress and Coping, investigators have found that nurse' mental health was negatively affected by COVID‐19 fear. A study conducted in the United States by Fitzpatrick et al. (2020) revealed a substantial positive correlation between the fear of COVID‐19 and various mental health outcomes, including increased symptoms of anxiety and depression, elevated stress level, and symptoms of post‐traumatic stress disorder. Khattak et al. (2021) in Pakistan reported similar findings for nurses, that is, a significant association between fear of COVID‐19 and high levels of anxiety, depression, and stress. Moghanibashi reported that 68.2% of the general population in Iran experienced moderate to severe levels of anxiety during the pandemic (Moghanibashi‐Mansourieh, 2020). In a systematic review, Xiong et al. (2020) confirmed the range of negative psychological consequences associated with the pandemic, that is, depression, stress, and insomnia. The aforementioned studies led us to hypothesize that fear of COVID‐19 was likely to have a substantial and detrimental effect on nurse mental health (Hypothesis 1).

### 1.2 The mediating effect of psychological capital

Nolzen (2018) proposed the concept of psychological capital, composed of self‐efficacy, hope, optimism, and resilience, to engender a positive outlook that augments personal and occupational success. Individuals who have little psychological capital may be particularly susceptible to the deleterious mental health effects stemming from the fear and ambiguity surrounding the COVID‐19 pandemic (Yu & Li, 2020). Ravikumar (2022) described how psychological capital functions as a protective mechanism against the detrimental influence of work‐related stress and anxiety. For example, greater levels of resilience corresponded with low levels of anxiety and fear linked to COVID‐19 (Li, 2020). Similarly, individuals with heightened self‐efficacy exhibit better coping abilities towards stress and uncertainty, thereby experiencing less fear and anxiety. Interventions targeted towards augmenting psychological capital are successful in enhancing mental health and well‐being in a variety of contexts (Song et al., 2019). In a systematic review, Burhanuddin et al. (2019) found that psychological capital interventions were notably efficacious in enhancing mental health. Furthermore, Da et al. (2020) reported that the efficacy of psychological capital intervention to enhance occupational attitudes and psychological resources of employees persisted for a 4‐month follow‐up period.

In summary, we postulate that psychological capital is a crucial mediator in the correlation between fear of COVID‐19 and mental health outcome (Hypothesis 2). By enhancing psychological capital, individuals may be better equipped to cope with pandemic‐induced stress and uncertainty, thereby maintaining their mental health.

### 1.3 The intermediary effect of burnout

Chronic exposure to work‐related stressors can lead to burnout, a complex psychological syndrome with manifestations of emotional exhaustion, depersonalization, and reduced personal accomplishment. Burnout is prevalent in healthcare professions, particularly nursing (Dall'Ora et al., 2020). Jun et al. (2021) reported a high likelihood of nurses experiencing burnout, which can have detrimental consequences. Fear of COVID‐19 promoted burnout. Ahorsuet et al. (2022) revealed that burnout and fear of COVID‐19 were positively correlated among nurses; nurses who experienced burnout were more likely to exhibit fear of COVID‐19, and vice versa. Individuals who develop burnout as a result of stressors typically exhibit a greater and more persistent display of negative emotions compared with persons who have experienced little or no burnout (Burić et al., 2019). However, for nurses with high levels of fear of COVID‐19, this lingering negative emotion is stronger (Fitzpatrick et al., 2020). The fear of COVID‐19 can intensify the subjective emotional effect of subsequent stress sources (Asmundson et al., 2020). Burnout, as chronic stress, can cause imbalances in neurotransmitters such as serotonin and dopamine (Yao et al., 2018), which may lead to a high level of fear of COVID‐19. Burnout and mental health are significantly negatively correlated (Hamed et al., 2020). Chronic stress always causes mental health problems and worsens life experiences. The more burnout, the more mental health problems (Pappa et al., 2021). Burnout heightens the experience of negative emotions, while also diminishing the ability to regulate negative information with coping strategies. This persistent negative state can lead to mental health risks (Bazargan‐Hejazi et al., 2021). Simultaneously, the presence of persistent mental health hazards can instigate a cyclical interaction between stress and mental health, thereby increasing the likelihood of a transition from short‐term to chronic mental health problems (Schneiderman et al., 2005). Individuals who exhibited elevated levels of fear of COVID‐19 were more likely to report having burnout (Abdelghani et al., 2020). Nurses exhibiting a high level of burnout are at an increased risk of developing mental health problems (Woon & Tiong, 2020). Consequently, heightened levels of fear of COVID‐19 are likely to result in an increased risk of burnout, which can negatively impact mental health. Thus, we posited that burnout is a mediating factor in the association between fear of COVID‐19 and mental health (Hypothesis 3).

### 1.4 The chain intermediary function of psychological capital and burnout

A strong negative correlation exists between psychological capital and burnout (Gong et al., 2019). Therefore, individuals who possess high levels of psychological capital are less likely to experience burnout (Li et al., 2019). Burnout can be mitigated by interventions based on psychological capital (Salanova & Ortega‐Maldonado, 2019). Biswal and Srivastava (2022) reported on individuals who underwent a psychological capital intervention for 28 days, involving eight 90‐min sessions of mindfulness‐based training. They found a statistically significant decrease in burnout following the intervention. A daily online self‐learning psychological capital intervention program fosters personal autonomy and control of the learning process, leading to long‐lasting changes in work‐related attitudes and diminished burnout (Da et al., 2020).

The core principles of psychological capital suggest that individuals with high levels of psychological capital are more likely to adopt a positive mindset towards their work and avoid dwelling on negative experiences and emotions, which ultimately limits burnout and enhances mental health. Thus, as Hypothesis 4, we posit that psychological capital and burnout are sequential mediators in the relationship between fear of COVID‐19 and mental health outcomes.

## 2 METHODS

We used a survey‐based cross‐sectional design to collect data from nursing personnel to examine the relations between fear of COVID‐19, psychological capital, burnout, and mental health.

### 2.1 Participants and procedure

We recruited nursing staff members from hospitals in Hefei, China, between 27 December 2022 and 5 January 2023. Recruitment was performed based on a comprehensive list of eligible candidates prepared by the administration of Hefei. An online questionnaire, administered by Wenjuanxing (https://www.wjx.cn/), was sent to 1000 staff members, of which 489 agreed to participate (48.9% response). To be eligible for inclusion in the study, participants must have had formal nursing education, current employment in a clinical department, and provision of online consent to participate. Participants who completed the 61‐item questionnaire in less than 180 s were excluded. Ultimately, data analysis was conducted on 445 participant samples.

### 2.2 Measures

#### 2.2.1 Fear of COVID‐19 scale

The level of fear of COVID‐19 was measured with the Fear of COVID‐19 Scale, developed by Ahorsu et al. (2022). The scale is composed of seven items having a five‐point Likert‐type response format from “strongly agree” (5) to “strongly disagree” (1). The total possible score, from 7 to 35, is the sum of the responses to the seven items. High scores indicate great fear of COVID‐19. The Chinese version of the Fear of COVID‐19 Scale was used for this study; the scale had an acceptable level of internal consistency (Cronbach's *α* = 0.92); (Chi et al., 2022).

#### 2.2.2 Psychological Capital

The Psychological Capital Scale was composed of 24 items, each rated on a 5‐point Likert scale from 1, disagree strongly to 5, agree strongly (Luthans et al., 2007). This scale consisted of four subdimensions, self‐efficacy, optimism, resilience, and hope. Lou et al. (2022) translated the scale into Chinese; the Cronbach *α* coefficients were 0.780–0.914.

#### 2.2.3 Burnout

The Maslach Burnout Inventory Human Services Survey for Medical Professionals was used to evaluate burnout (Erwan et al., 2020). Li et al. (2001) undertook the translation and revision of the scale into its Chinese version. The instrument consisted of 22 items, each with a frequency rating scale from “Never” (0) to “A few times a year or less” (1), “Once a month or less” (2), “A few times a month” (3), “Once a week” (4), “A few times a week” (5), and “Every day” (6). The tool assessed three areas, namely, Emotional Exhaustion, Depersonalization, and Sense of Personal Accomplishment. The scale employed in the present investigation had a Cronbach's *α* coefficient exceeding 0.82, which indicated reliable internal consistency.

#### 2.2.4 Mental health

To evaluate mental health, the Mental Component Summary of the 12‐item Short Form Health Survey (SF‐12) was administered (Ware Jr. et al., 1996). The Chinese version of the SF‐12 exhibited reliable internal consistency, as evidenced by a Cronbach's α coefficient exceeding 0.828 (Haitang et al., 2019).

### 2.3 Data analysis

Demographic data were summarized using descriptive statistics, including mean (standard deviation) and frequency (percent). To assess relations among variables, Pearson's correlation coefficient was implemented as a statistical measure. After verifying the correlations among variables by Pearson's correlation coefficient, we subjected a proposed mediation model (Figure 1) to analysis by the PROCESS macro (Hayes, 2017). The data were normalized using the *Z*‐score. The bootstrap method was used to evaluate the mediation effect, and the significance of the mediating effect was ascertained by examining whether the interval BootLLCI–BootULCI encompassed the value of 0. A non‐inclusive interval was indicative of a significant mediating effect, whereas an inclusive interval indicated a lack of significance in the mediating effect. Prior to conducting the data analysis, the Harman single‐factor test was administered to examine the presence of common method deviation. The findings of the study suggested the absence of any notable common method deviation. Specifically, the results of the Harman single‐factor test revealed that eight factors had characteristic roots greater than 1, collectively accounting for 69.517% of the total variance; the first factor explained 38.528% of the variation, which was below the recommended threshold of 40% (Podsakoff et al., 2003). SPSS version 26.0 was used for all statistical analyses.

## 3 RESULTS

### 3.1 Demographic characteristics

Following the predefined inclusion and exclusion criteria, we enrolled 445 nurses in this study. The average age of the study population was 34 years (SD = 6.76), with a predominance of women participants (*n* = 424; 95.20%).These demographic characteristics were similar to the characteristics of the entire nurse population in Anhui Province, in which the proportion of women nurses was 97.7% (National Health Commission of the People's Republic of China, 2020). A significant proportion of participants were married (*n* = 323; 72.5%) and had more than a decade of working experience (*n* = 238; 53.4%). Table 1 lists detailed demographic information.

**TABLE 1:** Participant characteristics (*N* = 445).

Variable	Mean ± SD or *n* (%)
Age (years)	32.89 ± 6.76
Sex (female)	424 (95.3%)
Years of experience	
0–5	114 (53.5%)
6–10	93 (20.9%)
>10	238 (53.5%)
Marital status	
Single	122 (27.4%)
Married	323 (72.6%)
Educational level	
College	41 (9.2%)
Bachelor	400 (89.9%)
Master	4 (0.9%)
Professional title	
Nurse	62 (13.9%)
Senior nurse	129 (29%)
Supervisor nurse	234 (52.6%)
Deputy chief nurse	20 (4.5%)

### 3.2 Pearson's correlation analysis

In Table 2, we report the Pearson product–moment correlation coefficients for the constructs of fear of COVID‐19, psychological capital, burnout, and mental health. Notably, the correlation coefficients, *r* = 0.315–0.632, all achieved statistical significance at a level of *p* < 0.01, thereby demonstrating a meaningful association between the analysed constructs.

**TABLE 2:** Pearson's correlation matrix of variables (*N* = 445).

Variables	Fear of COVID‐19	Psychological capital^b^	Burnout	MCS^a^
Fear of COVID‐19^d^	–	−0.315**	0.387**	−0.406**
Psychological capital^b^		–	−0.617**	0.516**
Burnout^c^			–	−0.632**
MCS^a^				–
Mean	20.320	87.910	28.060	41.260
SD	5.609	14.195	14.979	9.784

^a^ Assessed using the 12‐item Short Form Health Survey.

^b^ Assessed using Psychological Capital Scale.

^c^ Assessed using Maslach Burnout Inventory Human Services Survey for Medical Professionals.

^d^ Assessed using Fear of COVID‐19 Scale (Chinese version).

** *p* < 0.01.

### 3.3 Analysis of mediating effect

We used the statistical method (Zhonglin & Baojuan, 2014) to verify the intermediate effect. The mediating effect was evaluated using 5000 samples with estimation of the 95% confidence interval. Table 3 displays the results, which indicated that fear of COVID‐19 was a negative predictor of mental health (*β* = −0.406, *p* < 0.01), a negative predictor of psychological capital (*β* = −0.315, *p* < 0.01), and a positive predictor of burnout (*β* = 0.214, *p* < 0.01). Multivariate regression analysis, which included fear of COVID‐19, psychological capital, and burnout as independent variables, revealed that psychological capital had a positive effect on mental health (*β* = 0.182, *p* < 0.01), whereas burnout had a negative effect (*β* = −0.453, *p* < 0.01). Fear of COVID‐19 (*β* = −0.174, *p* < 0.05) had a statistically significant negative effect on mental health. It is important to note, however, that the strength of the relationship between fear of COVID‐19 and mental health was attenuated, with the coefficient β decreasing from −0.406 to −0.174.

**TABLE 3:** Regression results of the chain mediating effects model (*n* = 445).

Outcome variable	Predictive variable	*R* ^2^	*F*	*b*	SEs	*t*	LLCI	ULCI
Equation 1								
Psychological capital	Fear of COVID‐19	0.099	48.636**	−0.315**	0.045	−6.974	−0.403	−0.226
Equation 2								
Burnout	Fear of COVID‐19	0.422	161.355**	0.214**	0.038	5.623	0.139	0.289
	Psychological Capital			−0.550**	0.038	−14.427	−0.625	−0.475
Equation 3								
MCS	Fear of COVID‐19	0.451	120.648**	−0.174**	0.039	−4.517	−0.250	−0.098
	Psychological capital			0.182**	0.045	4.033	0.093	0.271
	Burnout			−0.453**	0.046	−9.756	−0.544	−0.362
Equation 4								
MCS	Fear of COVID‐19	0.165	87.588**	−0.406**	0.043	−9.359	−0.492	−0.321

*Note*: MCS Assessed using the 12‐item Short Form Health Survey. All estimated coefficients were not standardized. Number of bootstrap samples for percentile bootstrap confidence intervals is 5000.

Abbreviations: LLCI, Lower limit of the 95% CI; ULCI, Upper limit of the 95% CI.

** *p* < 0.01.

### 3.4 Comparison of chain mediating effect

**FIGURE 2:**
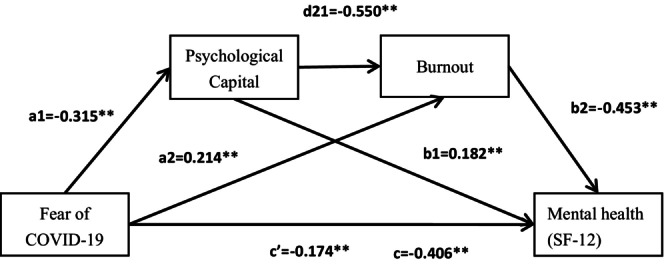
Chain mediating effect of fear of COVID‐19 and MCS. **p < 0.01. MCS Assessed using the 12‐Item Short Form Health Survey (SF‐12).

We found that the bootstrap 95% confidence interval of the total indirect effect of psychological capital and burnout on the association between fear of COVID‐19 and mental health did not encompass the index 0 (Table 4). The findings indicated that psychological capital and burnout had a statistically significant mediating effect on the relationship between fear of COVID‐19 and mental health. This mediating effect was composed of three paths, each of which was statistically significant. First, the path fear of COVID‐19 → psychological capital → mental health had a negative effect value of −0.057, which explained 14.078% of the total effect value, and the confidence interval for the interval did not include a value of 0. Second, fear of COVID‐19 → burnout → mental health demonstrated a substantial negative effect value of −0.097, accounting for 23.874% of the total effect value. Additionally, the confidence interval for the indirect effect did not contain a value of 0. Lastly, the path fear of COVID‐19 → psychological capital → burnout → mental health showed a negative effect value of −0.078, which explained 19.271% of the total effect value. The confidence interval for the indirect effect did not encompass a value of 0. Figure 2 shows the path model of the relation between fear of COVID‐19 and mental health.

**TABLE 4:** Results of chain mediating effect (*n* = 445).

	Effect	BootSE	BootLLCI	BootULCI	Ratio of indirect to total effect	Ratio of indirect to direct effect
Total effect	−0.4063	0.0434	−0.4916	−0.321	–	–
Direct effect	−0.1738	0.0385	−0.2495	−0.0982	–	–
Total indirect effect	−0.233	0.031	−0.295	−0.172	57.224%	133.774%
Ind1	−0.057	0.017	−0.094	−0.027	14.078%	32.911%
Ind2	−0.097	0.020	−0.138	−0.059	23.874%	55.811%
Ind3	−0.078	0.017	−0.114	−0.048	19.271%	45.052%

*Note*: MCS Assessed using the 12‐Item Short Form Health Survey (SF‐12). Ind1 is the mediation effect model of Fear of COVID‐19 → Psychological capital → MCS. Ind2 is the mediation effect model of Fear of COVID‐19 → Burnout → MCS. Ind3 is the mediation effect model of Fear of COVID‐19 → Psychological capital → Burnout → MCS. Boot SE, Boot LLCI, and Boot ULCL are estimated SE under bias‐corrected percentile bootstrap method. 95% CI lower and 95% CI upper, and Boot LLCI and Boot ULCL do not overlap with zero.

## 4 DISCUSSION

### 4.1 Mechanism of influence of fear of COVID‐19 on mental health

We measured the influence of fear of COVID‐19 on mental health in the nursing profession and assessed the mediating effect of psychological capital and burnout in the association between COVID‐19 fear and mental health. Fear of COVID‐19 had a statistically significant negative effect on nurse mental health, a finding that aligns with research by Labrague and de los Santos (Labrague & de los Santos, 2021). Our findings also demonstrate that psychological capital and burnout act as critical mediators in the link between fear of COVID‐19 and mental health. The direct predictive effect of fear of COVID‐19 on mental health was statistically significant when psychological capital and burnout were introduced as intermediary variables. In addition, both psychological capital and burnout exerted independent effects on mental health, and the chain of fear of COVID‐19 → psychological capital → burnout → mental health mediators also had statistically significant effects. Our results underscore the intricate association between fear of COVID‐19 and mental health in the nursing profession and the crucial function of psychological capital and burnout in mediating this relationship.

### 4.2 Mediating effect of psychological capital

Our findings suggest that fear of COVID‐19 can negatively affect the mental health of nurses by a distinct mediating effect of psychological capital. Nurses fearful of COVID‐19 may be susceptible to impairment of their psychological capital (Yaseen et al., 2021). High psychological capital means that nurses have confidence to move relentlessly towards goals; therefore, they do not fear COVID‐19 (Jeong & Kim, 2022). Enhancing the psychological capital of nurses can help them recover from adversity and increase their confidence, ultimately leading to a substantial improvement in their mental health (Kim & Kweon, 2020).

The discovery that fear of COVID‐19 can lead to a decrease in psychological capital and contribute to a decline in mental health aligns with findings of Ahorsu et al. (2022). Psychological capital can positively impact the mental health of nurses who experience high levels of COVID‐19‐related fear. Psychological capital, including self‐efficacy, optimism, hope, and resilience, can enhance an individual's confidence and lead to improved mental health outcomes.

### 4.3 Mediating effect of burnout

We found that burnout was a mediating agent between fear of COVID‐19 and mental health of nurses. First, nurses who experienced heightened levels of COVID‐19‐related fear may have encountered reinforced negative emotions and a subsequent rise in burnout. A recent systematic comparative review (Rizzo et al., 2023) shows differences between nurse burnout before and during the COVID‐19 pandemic. Pre‐COVID‐19 studies suggested moderate burnout, but the COVID‐19 era shows a notable increase in high emotional exhaustion and depersonalization, along with more frequent low scores for personal accomplishment. An increase in burnout can exacerbate the negative effect on mental health, ultimately leading to unfavourable outcomes (Ahorsu et al., 2022).

Second, a positive association existed between COVID‐19‐related fear and increased workplace stress, emotional exhaustion, and depersonalization symptoms. An increase in these symptoms can lead to heightened COVID‐19‐related fear (Raja et al., 2022). Our findings agree with a study of Chen and Meier (2021) that indicated a positive relationship between high burnout levels and increased likelihood of experiencing mental health issues. The COVID‐19 pandemic greatly affected the mental health of nurses, with fear of the virus and burnout emerging as crucial factors. Specifically, high levels of COVID‐19‐related fear increased burnout, which in turn worsened the mental health of nurses. Therefore, interventions that reduce burnout among nurses are crucial for promoting their mental well‐being.

### 4.4 The chain intermediary of nurse psychological capital and burnout

We found that COVID‐19‐related fear affected the mental health of nurses by the intermediary action of psychological capital and burnout. First, COVID‐19‐related fear can lower psychological capital, but, by addressing this fear and increasing psychological capital, one can reduce burnout and improve mental health. Çağış and Yıldırım (2023) reported that nurses who experienced high levels of fear of COVID‐19 tended to have significantly lower psychological capital while showing a statistically significant increase in burnout. By implementing an Inquiry‐Based Stress Reduction intervention program for 4 weeks, Zadok‐Gurman et al. (2021) observed a statistically significant improvement in the well‐being and resilience of teachers, and a noteworthy decrease in burnout amidst the challenging circumstances posed by the COVID‐19 pandemic. Zhang et al. (2020) reported similar findings. Therefore, it can be concluded that COVID‐19‐related fear may lead to a reduction in psychological capital, an increase in burnout, and ultimately a negative effect on the mental health of healthcare workers.

Previous studies establish a strong correlation between COVID‐19‐related fear and adverse mental health outcomes; however, few studies included assessing additional effects of internal psychological variables such as psychological capital and burnout on the mental health of nurses. This investigation has provided significant explanation of the interplay between COVID‐19‐related fear and mental health.

### 4.5 Implications for nursing/healthcare

Our findings hold noteworthy implications for promoting positive coping mechanisms among nurses in the face of the COVID‐19 pandemic. First, the fear of COVID‐19 by nurses may heighten their susceptibility to emotional exhaustion and a potential compromise of their mental well‐being. Therefore, it is essential for hospital administrators to prioritize their attention towards the fear of COVID‐19 and disseminate its significance. We recommend that hospitals organize various forms of psychological support activities to foster a positive culture and environment and to guide nurses in effective coping with the challenges posed by COVID‐19. Second, the key finding of the study highlights the chain intermediary effect, which suggests that a decrease in fear of COVID‐19, coupled with high amounts of psychological capital, may lead to reducing burnout and an improving overall mental health. Lupsa et al. (2019) showed that cognitive‐behavioural therapy, positive psychology interventions, and mindfulness‐based interventions were effective for enhancing psychological capital. Consequently, we recommend that mindfulness be included in nurse education courses and that efforts be made to enhance the psychological capital of nurses by various interventions.

### 4.6 Limitations and future directions

This study was a cross‐sectional survey; thus, data collection was limited to a single point in time with no follow‐up study. Such a limitation may impede establishing causal relationships between variables; thus, additional research based on longitudinal or experimental designs is needed to enable a thorough examination of the developmental process and key factors. Second, the questionnaire design and distribution were incomplete, which raised concerns about social acceptance factors influencing the responses. Additionally, the self‐report method employed to collect data may have been limited by time and location constraints, causing some degree of bias in the data. Third, the fear of COVID‐19 analysed in this study was only one of many factors that may influence mental health. Fourth, the study's exclusive focus on Chinese nurses limits the generality of the findings. Cultural and organizational variations across countries may impact nurses' experiences. To enhance external validity, further study should include samples from various geographical regions, facilitating the generalization of findings to a wider nursing population. Additionally, although we used the chain intermediary model to evaluate mediation, it is not the only mediating factor that could be considered. Variables such as job stress and workflow may contribute to mental health.

## 5 CONCLUSION

This study revealed a noteworthy negative correlation between fear of COVID‐19 and mental health outcomes, with psychological capital and burnout acting as significant mediators in the relationship. Specifically, there were three distinct mediating paths in the relationship between fear of COVID‐19 and mental health outcomes. These paths included the independent mediating effects of psychological capital and burnout and the chain mediating effect of psychological capital and burnout. This study showed that the fear of COVID‐19 may affect the mental health of nurses not only by the individual mediating influence of burnout but also by diminishing their psychological capital and intensifying burnout. Therefore, the findings indicate that fear of COVID‐19 does not directly cause mental health problems among nurses, but instead psychological capital and burnout act as crucial factors in the relationship.

Investigators have confirmed a strong link between the fear of COVID‐19 and heightened mental health problems. However, limited research has been conducted on the contribution of intrinsic psychological aspects, such as psychological capital and burnout. We addressed this gap by assessing how the fear of COVID‐19 affected the mental health of nurses and the extent to which this association was mediated by psychological capital and burnout. Our findings expand the theoretical scope of determinants of nurse mental health, and the findings provide evidence to hospital managers and nurses on the importance of strengthening psychological capital to ensure mental health.

## AUTHOR CONTRIBUTIONS

The study's conceptualization, data collection, and analysis, and manuscript preparation, were conducted by LCX and SNL. HC, JZ and MZ contributed to the manuscript's refinement. All authors have provided significant input to the article and have given their consent for the final version to be submitted for publication.

## FUNDING INFORMATION

The present research was supported by the Anhui Medical University 2020 Annual Research Fund (General Program; 2020xkj135) and the Anhui Medical University 2022 Annual Research Fund (General Program; 2022xkj311).

## CONFLICT OF INTEREST STATEMENT

The authors affirm that no actual or potential conflicts of interest exist in relation to this study, as it was conducted independently and without any commercial or financial affiliations.

## ETHICAL APPROVAL

The study adhered to ethical guidelines and was conducted in accordance with the principles outlined in the Declaration of Helsinki. The Institutional Review Board of the Second Affiliated Hospital of Anhui Medical University sanctioned the research protocol (SL‐YX2023‐093), which included the survey and the procedure for obtaining consent. Participants were assured of the confidentiality and anonymity of their responses, and the collected data were securely stored. All procedures complied with relevant national and international regulations regarding the protection of human research participants. The authors affirm their commitment to upholding the highest ethical standards throughout the research process.

## INFORMED CONSENT

Prior to participation in this study, all nursing staff members provided informed consent after receiving detailed information about the study's purpose, procedures, potential risks, and benefits.

## Data Availability

The datasets used in this study are available and can be retrieved from online repositories for public use (https://figshare.com/articles/dataset/data_export_csv/22647766).
